# Long non-coding RNA NEAT1 regulates epithelial membrane protein 2 expression to repress nasopharyngeal carcinoma migration and irradiation-resistance through miR-101-3p as a competing endogenous RNA mechanism

**DOI:** 10.18632/oncotarget.19596

**Published:** 2017-07-26

**Authors:** Yujia Wang, Chunting Wang, Can Chen, Fengbo Wu, Pengfei Shen, Peng Zhang, Gu He, Xiang Li

**Affiliations:** ^1^ Department of Urology and State Key Laboratory of Biotherapy, West China Hospital, West China Medical School, Sichuan University, Chengdu 610041, P.R. China; ^2^ Department of Radiation Oncology, Sichuan Cancer Hospital & Institute, Sichuan Cancer Center, School of Medicine, University of Electronic Science and Technology of China, Chengdu 610041, P.R. China; ^3^ School of Pharmacy, Chengdu Medical College, Chengdu 611137, P.R. China

**Keywords:** NEAT1, EMP2, miR-101-3p, nasopharyngeal carcinoma, sponge

## Abstract

The altered expression of long non-coding RNAs (lncRNAs) is often related to carcinogenesis, metastasis and resistance to radiation or chemotherapy. In the current study, cDNA microarray analysis found that NEAT1 expression was reduced in nasopharyngeal carcinoma (NPC) patients and that it regulated NPC progression. However, the detailed mechanisms of NEAT1 in NPC were unclear. NEAT1 repressed NPC cell growth, invasion and radiation resistance *in vitro* and tumor metastasis *in vivo*. In addition, the results of an approach integrating bioinformatics, luciferase reporter assays and RNA immunoprecipitation indicated that NEAT1 antagonized miR-101-3p through a competing endogenous RNA (ceRNA) mechanism and that the interaction between NEAT1 and EMP2 was miR-101-3p dependent. Our results showed a novel connection of NEAT1, miR-101-3p and EMP2 in NPC migration and radiation resistance.

## INTRODUCTION

Nasopharyngeal carcinoma (NPC) is among the most common carcinomas originating in the head and neck region and is the third leading cause of cancer-related death worldwide [1]. NPC is a cancer showing strong ethnic and geographic tendencies. The annual incidence of NPC in south China constituted approximately 80% of cases in the whole country and approximately 65% of worldwide incidences [2]. Many risk factors contribute to NPC pathogenesis, among which Epstein-Barr virus (EBV) infection plays an important role [3–5]. Moreover, NPC is associated with a panel of genetic and epigenetic alterations, such as single-nucleotide polymorphisms (SNPs), familial aggregation, DNA methylation, microRNA and long non-coding RNA (lncRNA), among others [6–9]. The main therapeutic method for NPC is irradiation therapy, alone or combined with chemotherapy. Irradiation therapy may prolong survival even in advanced NPC. However, radiation resistance remains a major obstacle to successful treatment of advanced NPC. The detailed mechanism by which NPC develops radiation resistance and metastatic recurrence remains unclear [10–13]. Hence, the identification of novel prognostic biomarkers and therapeutic targets is urgently needed for NPC patients.

lncRNAs are a subgroup of non-coding RNAs with lengths greater than 200 bps. Various studies have shown that lncRNAs can regulate gene expression levels and post-transcriptional modifications; bind to transcription factors, mRNAs or miRNAs; and play modulator roles in many biological processes [14–19]. Notably, the dysfunction of lncRNAs has also resulted in abnormal gene regulation and contributes to the carcinogenesis, progression, metastasis and resistance to radiation or chemical therapeutics of NPC and other tumors. A number of dysregulated lncRNAs are involved in various biological activities of NPC, e.g., HOTAIR, ANRIL, MALAT1, AFAP1-AS1, AL359062, NEAT1, EWSAT1, MEG3, HNF1A-AS, H19 and ROR [20–38]. Recently, interactions between mRNA and lncRNA that occur through competitive binding to shared miRNA sponges have been found to be a novel regulation mechanism of lncRNAs [14, 39–41]. However, the detailed biological functions and clinical significance of many differentially expressed lncRNAs and their regulatory network in the carcinogenesis, metastasis and radio-resistance of NPC remain unclear.

In this study, we profiled differentially expressed lncRNAs from three previously published NPC cohorts (GSE13597, GSE64634 and GSE12452) [32, 42, 43]. Nuclear enriched abundant transcript 1 (NEAT1) was identified as a potential prognostic marker and an important regulator for the maintenance of prostate cancer. The expression profile of NEAT1 was determined in paraffin-embedded NPC tissue specimens. Over-expression and knockdown experiments targeting NEAT1 both *in vitro* and *in vivo* were performed to assess the alteration of NPC cell behaviors when the NEAT1 level was increased or decreased. Our study provides a significant step toward an in-depth understanding of the functions and regulatory mechanisms of lncRNAs and offers novel insights into the interactions of NEAT1 and EMP2 in the radio-resistance and migration of NPC cells. Future studies based on these findings may lead to the discovery of novel NPC biomarkers or targeted therapies. We hope that NEAT1-targeted therapeutics might be developed as a potential therapeutic strategy, alone or combined with irradiation, for the diagnosis and treatment of nasopharyngeal carcinoma.

## RESULTS

### NEAT1 is down-regulated in NPC tissues and is associated with poor prognosis

**Figure 1:**
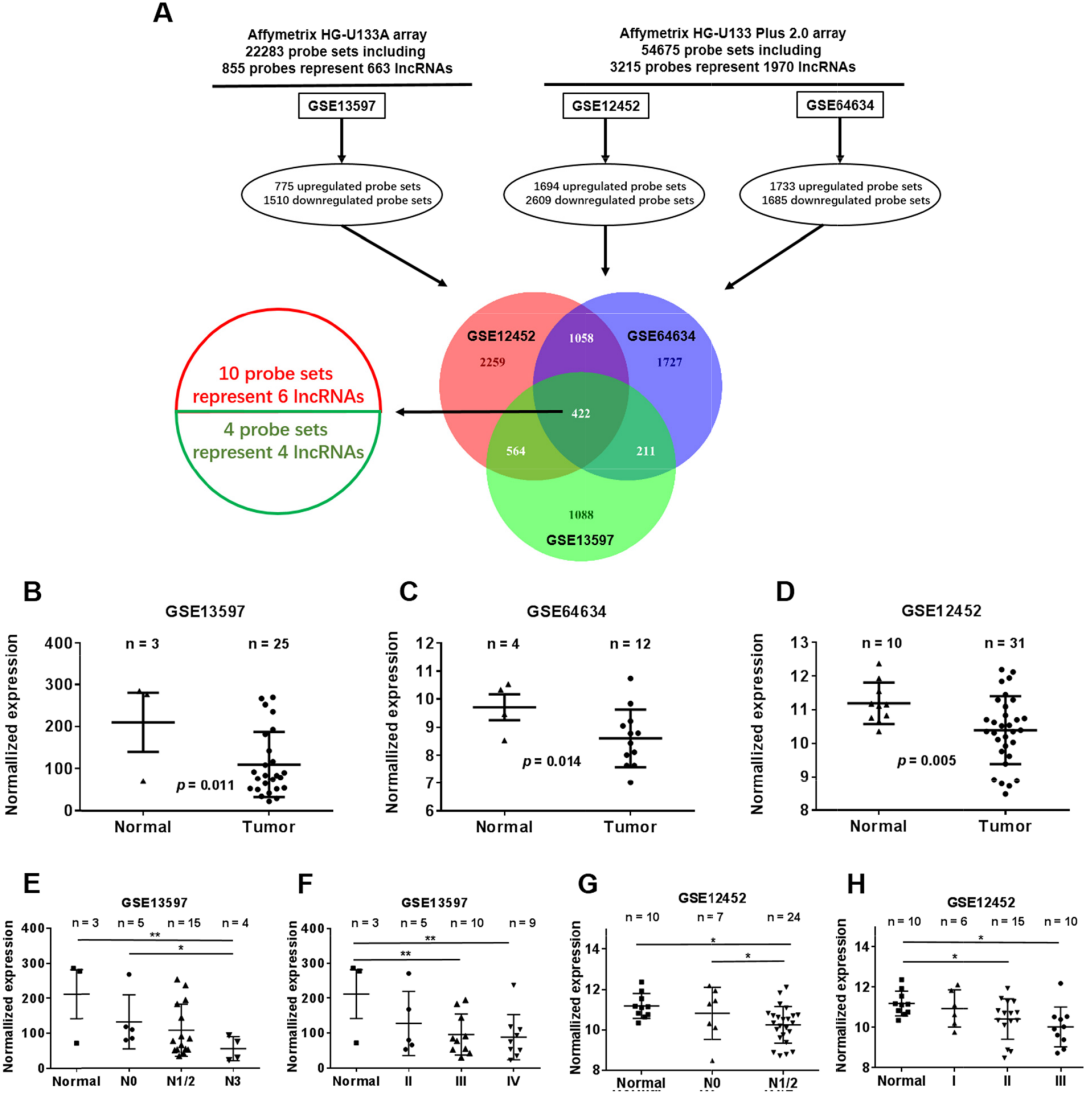
Dysregulated lncRNA expression analysis use three independent NPC cohorts **(A)** Schematic overview of the workflow used to identify and validate dysregulated lncRNAs in three NPC microarray data cohorts. **(B)** NEAT1 expression, as measured by Affymetrix Microarray, was down-regulated in NPC biopsies (T, n = 25) when compared with normal nasopharyngeal epithelia tissues (N, n = 3). **(C)** Down-regulated NEAT1 expression was confirmed in NPC biopsies (T, n = 12) compared with non-tumor NPE tissues (N, n = 4). **(D)** NEAT1 expression, as measured by Affymetrix Microarray, was down-regulated in NPC biopsies (T, n = 31) when compared with non-tumor NPE tissues (N, n = 10). **(E)** NEAT1 expression was associated with lymph node metastasis in the GSE13597 dataset (normal: non-tumor NPE; NPC_N0: NPC biopsies without lymph node metastasis; N1/2: NPC biopsies with lymph node metastasis; N3: NPC biopsies with lymph node metastasis>6cm). **(F)** NEAT1 expression was associated with clinical disease stages in the GSE13597 dataset (normal: non-tumor NPE; II, III or IV: NPC biopsies with clinical stage II, III or IVdisease). **(G)** NEAT1 expression was associated with lymph node metastasis in the GSE12452 dataset (normal: non-tumor NPE; NPC_N0: NPC biopsies without lymph node metastasis; N1/2: NPC biopsies with lymph node metastasis;). **(H)** NEAT1 expression was associated with clinical disease stages in the GSE12452 dataset (normal: non-tumor NPE; I, II or III: NPC biopsies with clinical stage I, II or III disease).

We first profiled differentially expressed genes in NPC and normal nasopharyngeal epithelia tissues (GSE12452, GSE64634 and GSE13597). These three NPC gene expression cohorts were analyzed using the Affymetrix HG-U133A array and the Affymetrix HG-U133 Plus 2.0 array. We found 2285 differentially expressed genes in the GSE13597 dataset, 4303 in the GSE12452 dataset and 3418 in the GSE64634 dataset. Based on NetAffx, Refseq and Ensembl non-coding RNA annotations, we identified 14 overlapping probe sets representing 10 lncRNAs that were differentially expressed in NPC compared to normal nasopharyngeal epithelia. Of these ten lncRNAs, six were up-regulated, and four were down-regulated (Figure 1A). All things considered, we chose NEAT1 for the follow-up study.

**Figure 2:**
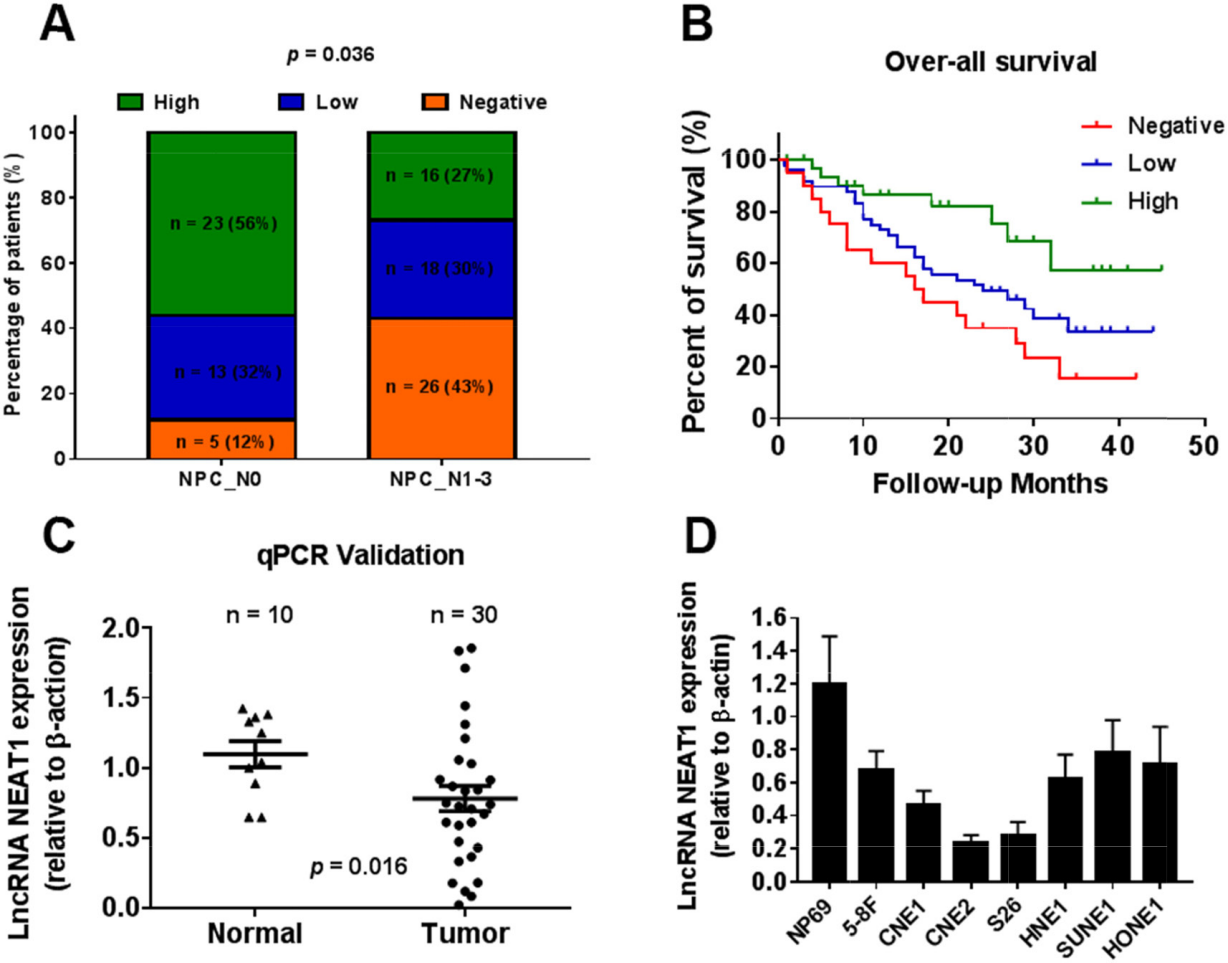
Down-regulated NEAT1 is associated with tumor metastasis and poor prognosis **(A)** Proportion of NPC patients with NEAT1 expression with lymph node metastasis (N0, n = 10; N1-3, n = 30, p = 0.036). **(B)** The Kaplan-Meier survival analysis indicated that NEAT1 high expression (green line) has a worse overall survival compared to the low expression (blue line) and negative (red line) subgroup. **(C)** NEAT1 expression in non-tumor NPC tissues (N, n = 10) and NPC biopsies (T, n = 30) was validated in another cohort of NPC samples using qRT-PCR(p=0.016). **(D)** Relative expression of NEAT1 expression in NPC cell lines and normal nasopharyngeal epithelial cell NP69.

Among the differentially expressed lncRNAs, NEAT1 was highly depressed in the NPC samples of all datasets (GSE13597, GSE64634 and GSE12452). The expression of NEAT1 was lower in tumor tissues than in normal tissues (Figure 1B-1D). Low NEAT1 expression was associated with a number of clinicopathological parameters in the GSE13597 and GSE 12452 datasets, including lymph node metastasis and TNM stage. It was shown that the expression of NEAT1 is associated with lymph node metastasis rather than TNM stage (*p<0.05, **p<0.01) (Figure 1E-1H). We then analyzed NEAT1 expression for associations with clinicopathological parameters, such as gender, age, smoking, histological type, pathological stage, tumor size (T stage), lymph-vascular invasion (N stage) and relapse. The data indicated that NEAT1 expression had a non-significant association with advanced tumor stage (Figure 2A). In addition, NPC patients with high NEAT1 expression levels (>2-fold actin) had longer overall survival than patients with low (0.2∼2 fold actin) and negative (<0.2-fold actin) NEAT1 expression levels (Figure 2B), as indicated by Kaplan-Meier survival analysis (Figure 2B). These results demonstrated that low expression levels of NEAT1 were associated with poor prognosis. To verify if NEAT1 was differentially expressed in NPC tissues, 30 NPC tissues and 10 adjacent NPC tissues were tested for NEAT1 expression. As expected, NEAT1 expression was markedly decreased in NPC tissues compared to their normal counterparts (Figure 2C). Further, we detected NEAT1 expression in cell lines, and the results indicated that NEAT1 expression was lower in NPC cell lines, including 5-8F, CNE1, CNE2, S26, HNE1, SUNE1, and HONE1, than in normal NP69 (nasopharyngeal epithelial) cells (Figure 2D). These data demonstrated that the down-regulation of NEAT1 may play important roles in NPC development and progression.

### NEAT1 suppresses migration in NPC cells but has a limited effect on cell proliferation

**Figure 3:**
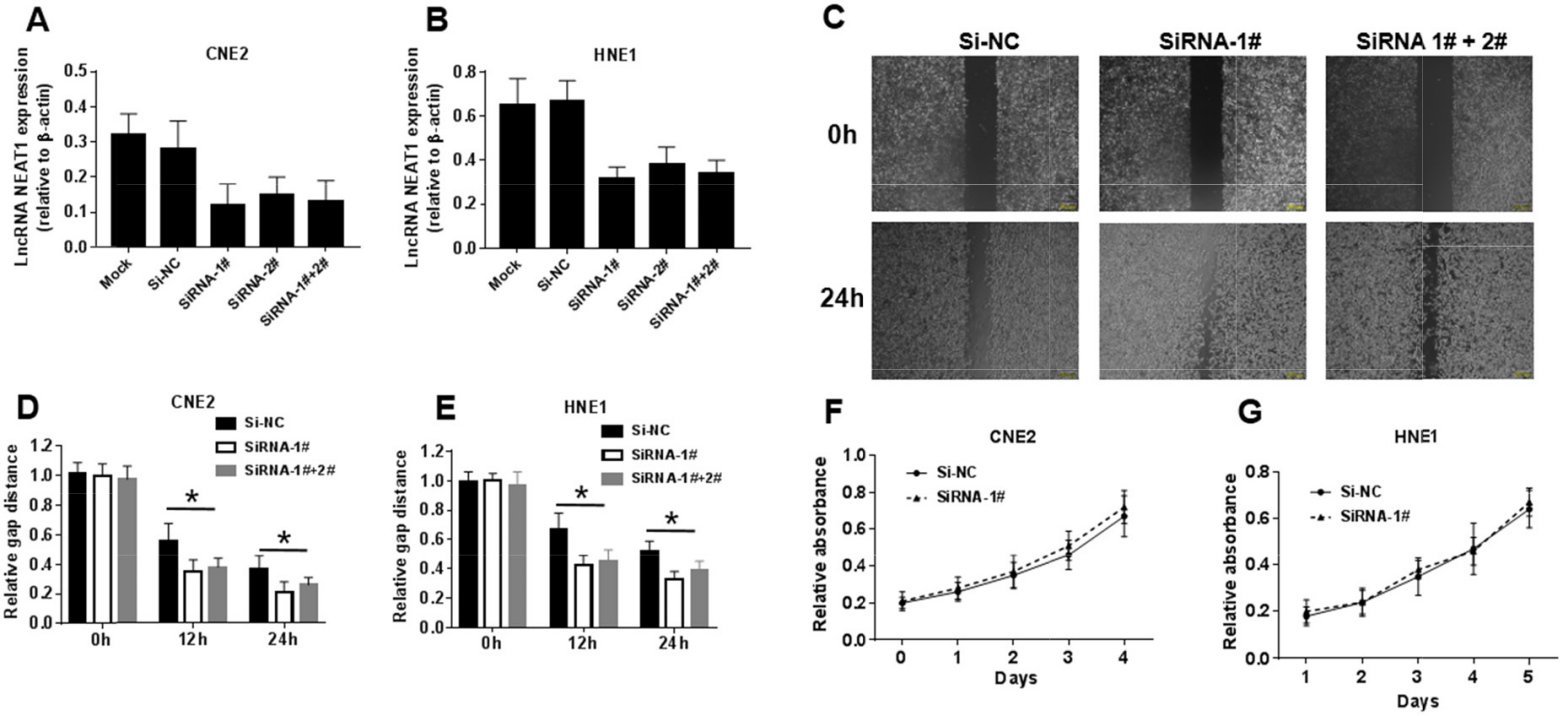
NEAT1 suppresses migration in NPC cells but has its limitation in cell proliferation **(A-B)** NEAT1 expression levels in different siRNA transfected group. **(C-E)** Effects of NEAT1 on cell migration in CNE2 and HNE1 NPC cells analyzed using Wound-healing assay. The data represent the mean ± S.D. of three different experiments. *p< 0.05 versus scramble control. **(F-G)** Effects of NEAT1 on cell proliferation in CNE2 and HNE1 NPC cells analyzed using the MTT assay.

To investigate the role of NEAT1 in NPC cells, CNE2 and HNE1 NPC cells were transiently transfected with siRNA-1#, siRNA-2# and siRNA-1#+2#. The results demonstrated that both siRNA oligonucleotides could efficiently knock down NEAT1 expression in each of these cell lines. The lncRNA NEAT1 expression of NPC cells transfected with siRNA-1#, siRNA-2# and siRNA-1#+2# was 50% lower than for NPC cells transfected with si-NC and Mock (Figure 3A-3B). Because siRNA-1# had a better silencing effect on NEAT1 than siRNA-2#, and there was no difference in effect between siRNA-1# and siRNA-1#+2#, we used siRNA-1# for further research. After establishing siRNA efficacy, we assessed the phenotype changes induced by NEAT1 knockdown in the NPC cell lines. The wound healing assay showed that low NEAT1 expression resulted in considerably faster migration in both cell lines. After 12 h and 24 h, the relative gap distance of NPC cells transfected with siRNA-1# and siRNA-1#+2# was 30% narrower than that for NPC cells transfected with si-NC (Figure 3C-3E). The MTT assay showed that NEAT1 had no obvious effects on cell growth (Figure 3F-3G). Collectively, these results indicated that NEAT1 strongly inhibited migration but had no obvious effect on cell proliferation.

### NEAT1 can enhance radiosensitivity and promote cell apoptosis in NPC cells

**Figure 4:**
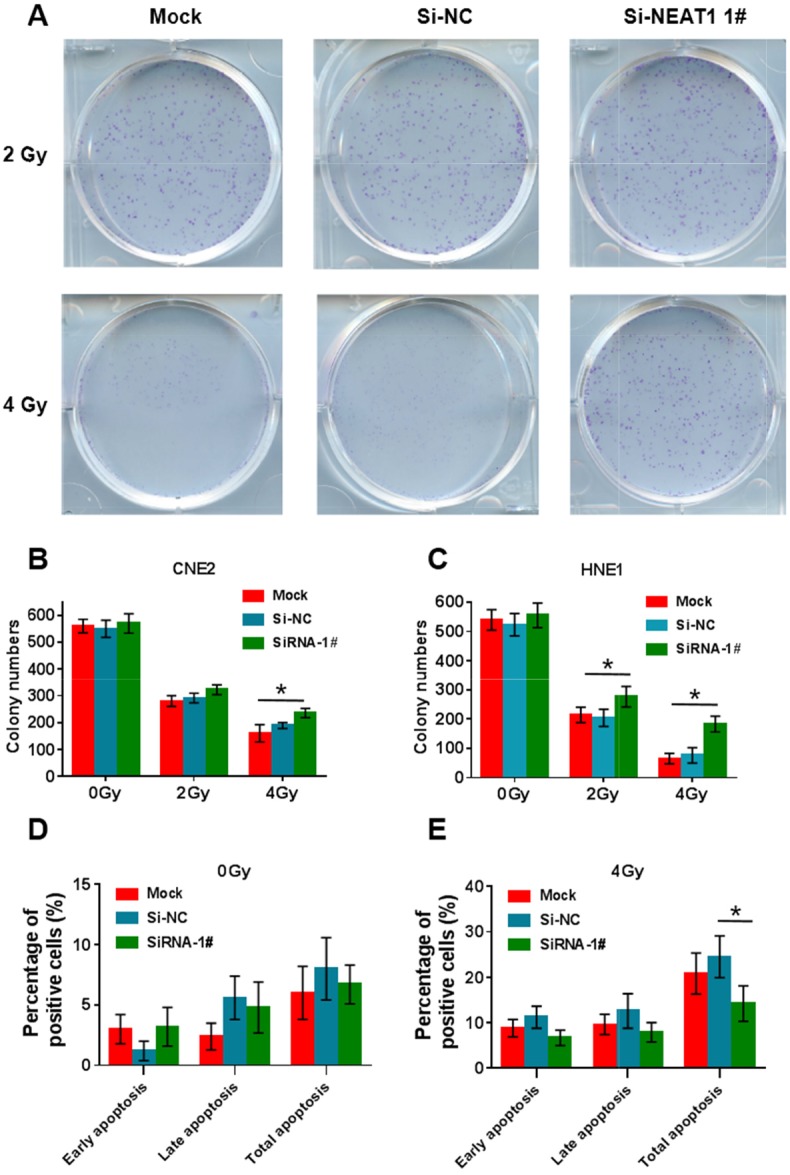
NEAT1 can enhance radiosensitivity and promote cell apoptosis in NPC cells **(A-C)** After radiation treatment, CNE2 and HNE1 cells with si-NEAT1 were subjected to colony formation assay and statistical analysis. As shown, a significant increasing in colony number was observed in si-NEAT1 group under 4Gy radiation condition (*: p<0.05). **(D-E)** si-NEAT1 combined with radiation treatment depresses cell apoptosis in CNE2 cells. Flow cytometer assays were performed to examine cell apoptosis by annexin V-FITC/propidium iodide (PI) double staining. si-NEAT1 combined with 4 Gy radiation treatment significantly depresses cell apoptosis in CNE2 cells. Early apo, early stage apoptosis; Late apo, late stage apoptosis; Total apo, both late and early stage apoptosis (*: p<0.05).

To examine whether NEAT1 can enhance radiosensitivity in NPC cells, CNE2 and HNE1 cells with NEAT1 silenced were subjected to colony formation assays (Figure 4A-4C). As shown, a significant increase in colony number was observed in the si-NEAT1 group under 4 Gy radiation, which demonstrated that NEAT1 can enhance radiosensitivity significantly in NPC cells. We further examined whether si-NEAT1 combined with radiation treatment can depress cell apoptosis by annexin V-FITC/propidium iodide (PI) double staining and flow cytometry (Figure 4D-4E). As shown, si-NEAT1 combined with 4 Gy radiation treatment significantly depressed both early-stage and late-stage cell apoptosis in CNE2 cells.

### miR-101-3p can directly bind to NEAT1 at the miRNA recognition site

**Figure 5:**
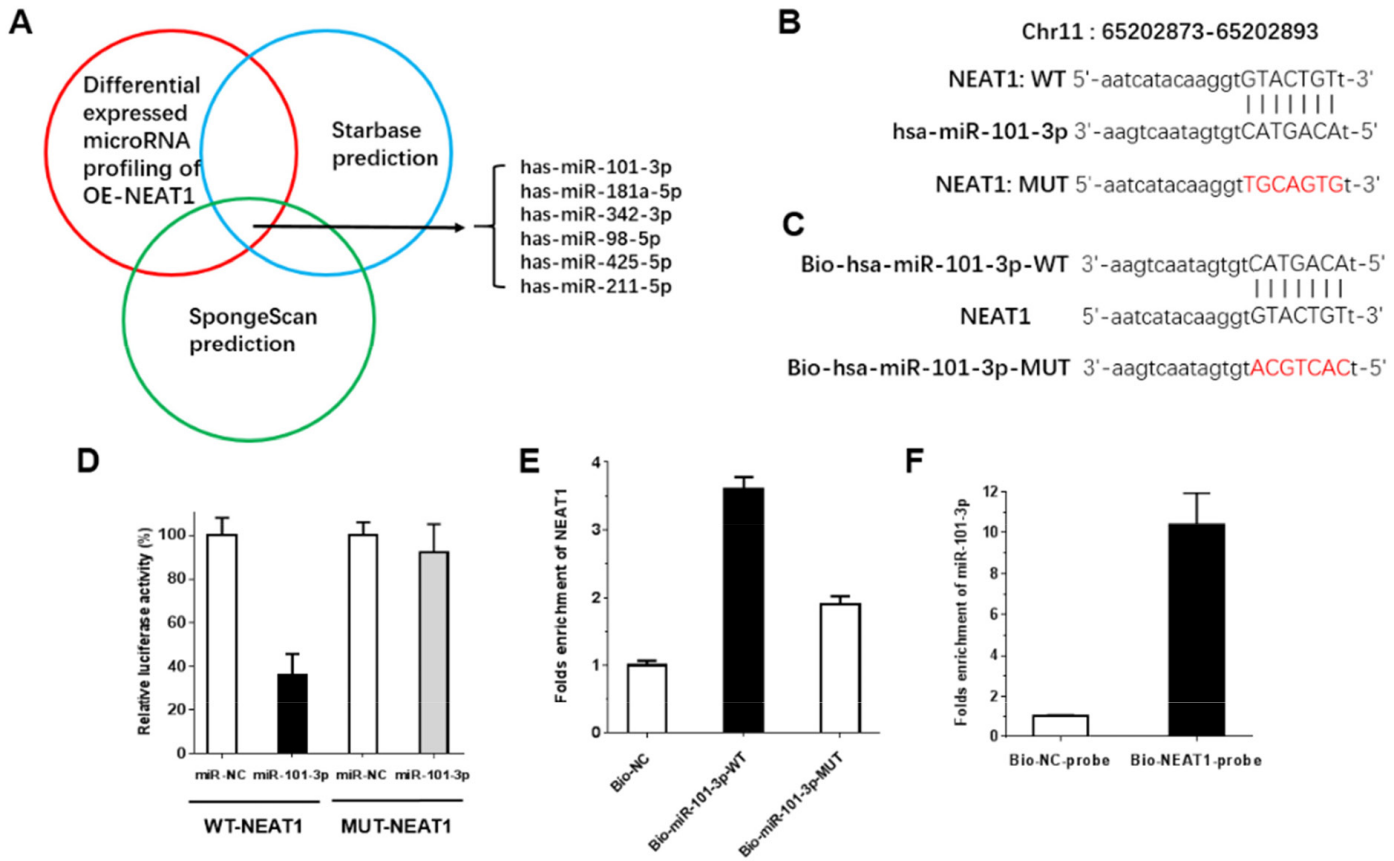
NEAT1 is direct target of miR-101-3p **(A)** Screen of the candidate miRNAs that interacted with OE-NEAT1 by real-time PCR-based miRNA expression profiling and starBase (v2.0) and SpongeScan. Co-analysis of the up-regulated miRNAs in stable overexpression NEAT1 in CNE2 cells compared to the control CNE2 cells and the miRNA list that potentially target NEAT1 predicted by starBase (v2.0) and SpongeScan, shown in Figure 5A, we got six candidates. **(B-D)** The predicted miR-101-3p binding sites in the e 3′-UTR region of NEAT1 (WT-NEAT1) and the designed mutant sequence (MUT-NEAT1) are indicated. Cell were transfected with MUT-NEAT1 and the indicated miRNAs, and then the Luciferase reporter assay was conducted. Data are presented as the mean ± S.D. (n = 5, each group). **(E)** CNE2 cells transfected with biotinylated WT miR-101-3p (Bio-miR-101-3p-WT) or biotinylated mutant miR-101-3p (Bio-miR-3p-MUT) or biotinylated NC (Bio-NC), assayed by biotin-based pulldown 48 h after transfection. NEAT1 levels were analyzed by RT–qPCR. **(F)** CNE2 cells transfected with biotinylated NEAT1 probe (Bio-NEAT1-probe) or biotinylated NC probe (Bio-NC-probe), assayed by biotin-based pulldown 48 h after transfection. MiR-101-3p levels were analyzed by RT–qPCR.

Increasing numbers of publications have demonstrated that lncRNAs can act as a molecular sponge or a ceRNA in regulating the accumulation of miRNA, in turn affecting its biological functions. StatBase2.0 and spongeScan were used to predict and select miRNAs that interact with NEAT1. Moreover, qRT-PCR-based miRNA profiling was used to select the differentially expressed miRNAs in OE-NEAT1. Integrating the prediction by bioinformatics and the verification by miRNA profiling analysis, miR-101-3p, miR-181a-5p, miR-342-3p, miR-98-5p, miR-425-5p and miR-211-5p were selected as candidate miRNAs (Figure 5A). We chose miR-101-3p to verify the relationship between NEAT1 and a candidate miRNA. To further investigate whether NEAT1 was a functional target of miR-101-3p, a dual-luciferase reporter assay was performed after mutation. NEAT1 was predicted to harbor one miR-101-3p binding site. Our results showed that the luciferase activity was significantly decreased by co-transfection with miR-101-3p and WT-NEAT1 compared to co-transfection with miR-NC and WT-NEAT1, suggesting that NEAT1 was the target of miR-101-3p. Meanwhile, co-transfection with miR-101-3p and MUT-NEAT1 did not change the luciferase activity, suggesting that the miR-101-3p binding site within NEAT1 was functional (Figure 5B-5D). miRNAs function through RNA-induced silencing complex (RISC). Ago2 is an essential catalytic component of RISC involved in RNA cleavage. We performed pull-down experiments using biotin-labeled miR-101-3p oligos. NEAT1 was pulled down by biotin-labeled miR-101-3p oligos but not by the mutated oligos (binding sites were mutated to the complementary sequences) or biotin-labeled NC (Figure 5E). In addition, we performed an inverse pulldown assay using a biotin-labeled specific NEAT1 probe to verify whether NEAT1 could pull up miR-101-3p, and miR-101-3p was precipitated and analyzed by q-RT-PCR analysis (Figure 5F). It was shown that NEAT1 could pull up miR-101-3p remarkably well. These results revealed that miR-101-3p can directly bind to NEAT1 at the miRNA recognition site.

### NEAT1 can enhance radiosensitivity and is suppressed by miR-101-3p

**Figure 6:**
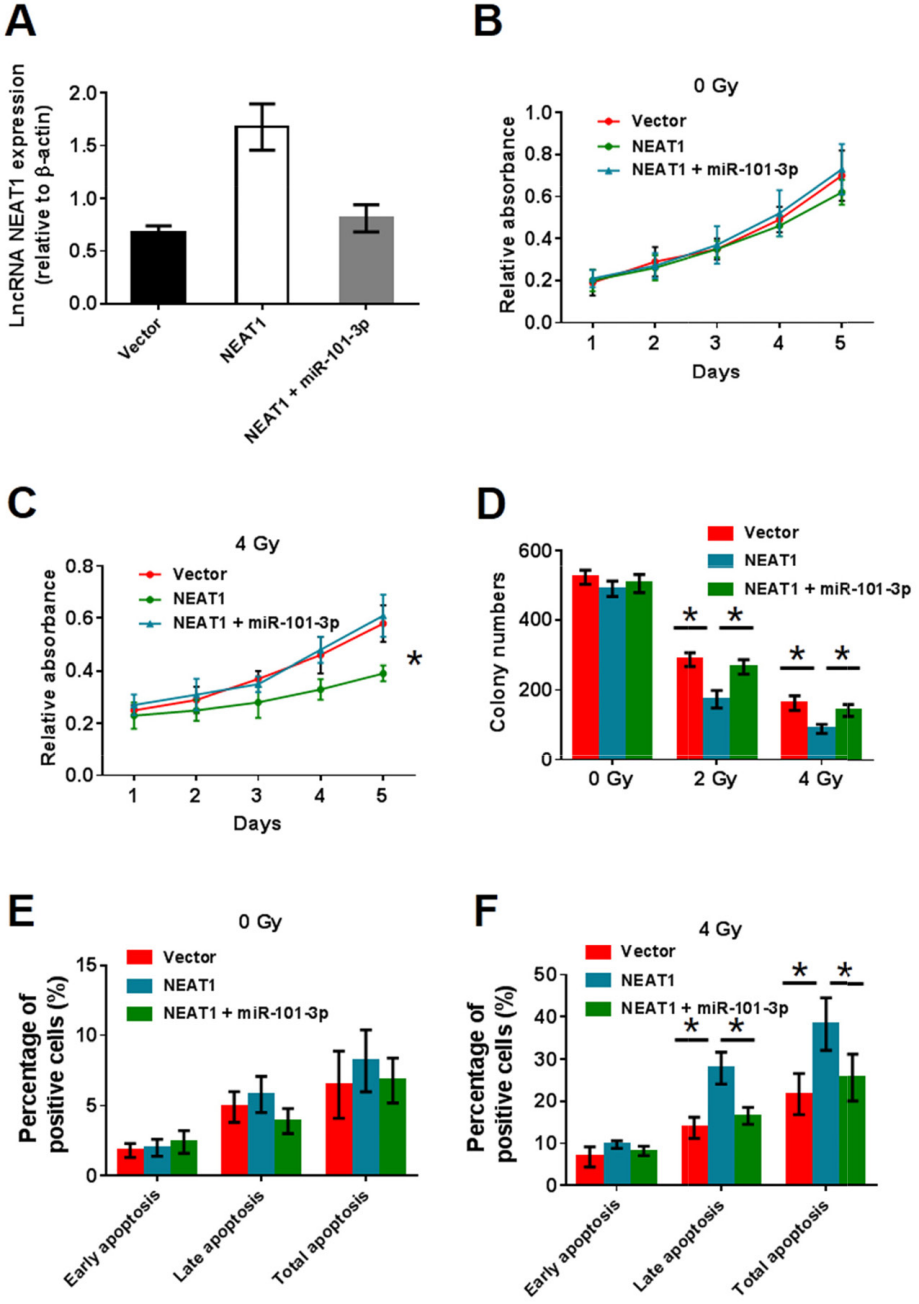
NEAT1 can enhance radiosensitivity and miR-101-3p has a competition with NEAT1 **(A)** NEAT1 expression in vector, OE-NEAT1 and OE-NEAT1+miR-101-3p group. **(B-C)** Effects of NEAT1 and NEAT1+miR-101-3p over-expression on cell proliferation in CNE2 and HNE1 NPC cells analyzed using the MTT assay. (**D)** After radiation treatment, HNE1 cells with NEAT1 and NEAT1+miR-101-3p over-expression were subjected to colony formation assay and statistical analysis. As shown, a significant decreasing in colony number was observed in NEAT1group under 2Gy and 4Gy radiation condition. However, NEAT1+miR-101-3p group has no difference with vector (*: p < 0.05). **(E-F)** NEAT1+miR-101-3p over-expression combined with radiation treatment depresses cell apoptosis in CNE2 cells. Flow cytometer assays were performed to examine cell apoptosis by annexin V-FITC/propidium iodide (PI) double staining NEAT1+miR-101-3p over-expression combined with 4Gy radiation treatment significantly depresses both early stage and late stage cell apoptosis in CNE2 cells (*: p < 0.05).

Although the interaction between miR-101-3p and NEAT1 was confirmed, the biological behaviors of NPC cells regulated by miR-101-3p and NEAT1 still needed to be determined. To investigate the role of NEAT1 and miR-101-3p in NPC cells, NEAT1 was over-expressed in CNE2. We compared the OE-NEAT1 group with the OE-NEAT1+miR-101-3p group. Q-RT-PCR analysis showed that the expression of NEAT1 in the vector and OE-NEAT1+miR-101-3p group was significantly lower than in the OE-NEAT1 group (Figure 6A). To examine whether the OE-NEAT1+miR-101-3p group could enhance radiosensitivity in NPC cells, we performed MTT and colony formation assays (Figure 6B-6D). As shown, a significant decrease in relative absorbance was observed in the OE-NEAT1 group under 4 Gy radiation, and miR-101-3p reversed the decrease. At the same time, a significant reduction in colony numbers was observed in the OE-NEAT1 group under 2 Gy and 4 Gy radiation, and miR-101-3p reversed the decrease. We further examined whether OE-NEAT1 combined with radiation treatment could induce cell apoptosis by annexin V-FITC/propidium iodide (PI) double staining and flow cytometry (Figure 6E-6F). As shown, the over-expression of NEAT1 combined with 4 Gy radiation treatment significantly promoted both early-stage and late-stage cell apoptosis in CNE2 cells, while miR-101-3p could reverse the increase in apoptosis. In conclusion, NEAT1 can enhance radiosensitivity, while miR-101-3p competes with NEAT1.

### EMP2 is the target of miR-101-3p

**Figure 7:**
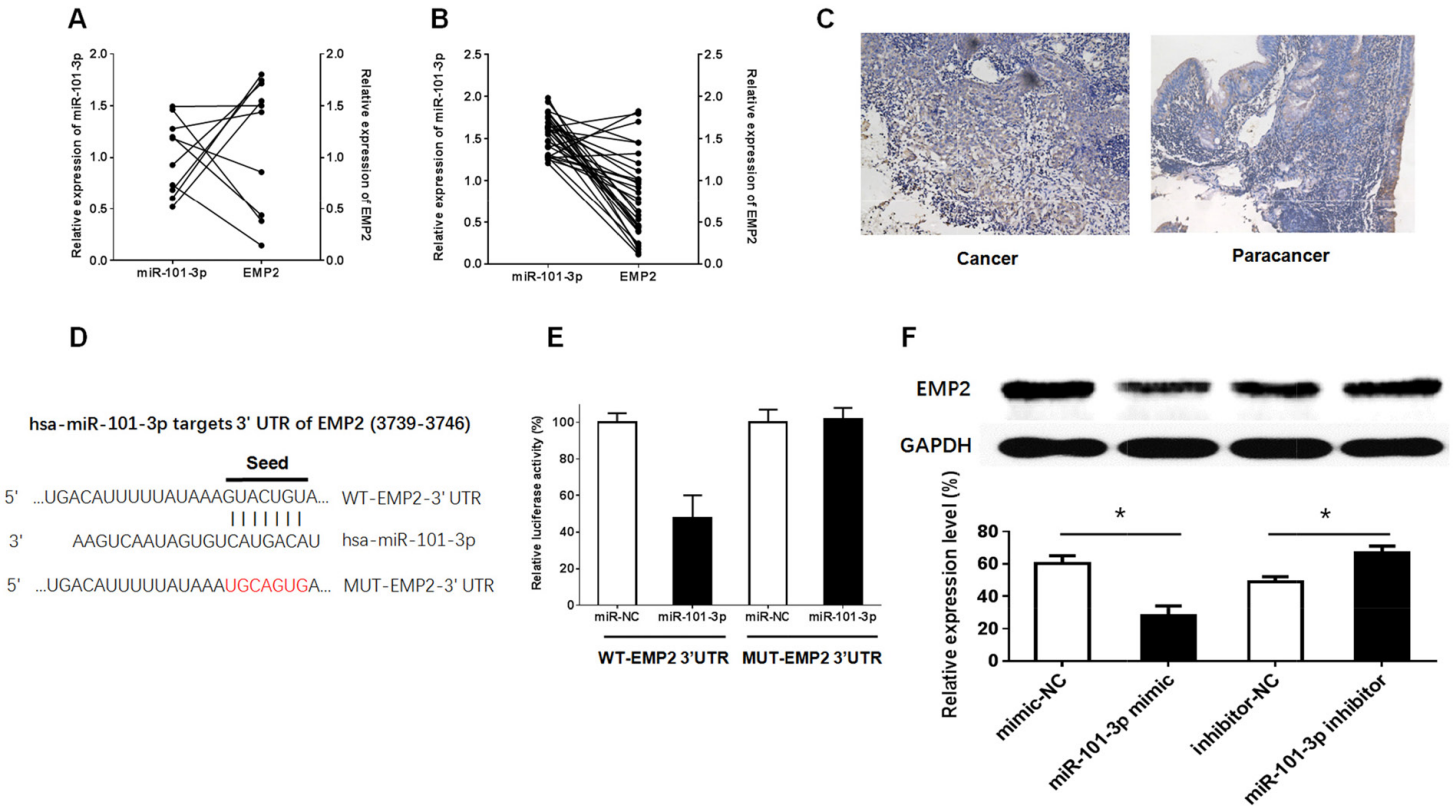
EMP2 was the target of miR-101-3p **(A-B)** The relationship between relative expression of miR-101-3p and relative expression of EMP2 in 30 patients of NPC tissues and in 10 normal nasopharyngeal epithelia tissues. The average NEAT1 expression was normalized by β-actin expression (**: p < 0.01, ***: p < 0.001). Relative expression was analyzed by the 2-ΔCT method normalized to β-actin. Experiments were performed in triplicate, and the error bars represent S.D. for all panels. **(C)** Immunohistochemistry of EMP2 in tumors isolated from cancer tissues and paracancer tissues. (**D-E)** The predicted miR-101-3p binding site of 3’UTR of EMP2(WT-EMP2 3’UTR) and the designed mutant sequence (MUT-EMP2 3’UTR) are indicated. Cells were transfected with WT-EMP2 3’UTR (or WT-EMP2 3’UTR) and the indicated miRNAs, and then the luciferase reporter assay was conducted. Data are presented as the mean ± S.D. (n = 5, each group). **(F)** Effects of miR-101-3p on the expression of EMP2 in the protein levels. Data are presented as the mean ± S.D. (n = 2, each group) (*: p < 0.05).

EMP2 re-expression significantly suppressed cell growth and promoted radiosensitivity and cell apoptosis in CNE-2 cells. Thus, EMP2 is down-regulated and serves as a potential tumor suppressor gene in NPC cells [44]. In parallel, as shown in Figure 7A-7B, q-RT-PCR showed that miR-101-3p down-regulates EMP2 in NPC tissues; however, there is no relationship between miR-101-3p and EMP2 in normal nasopharyngeal epithelial tissues. Next, immunostaining analysis of EMP2 was performed in NPC cancer tissues and paracancer tissues. The number of EMP2-positive cells was significantly higher in paracancer tissues groups than in NPC cancer tissues (Figure 7C). To further investigate whether EMP2 is a functional target of miR-101-3p, a dual-luciferase reporter assay was performed. EMP2 was predicted to harbor one miR-101-3p binding site. Our results showed that the luciferase activity was significantly decreased by co-transfection with miR-101-3p and WT-EMP2 3’UTR compared to co-transfection with miR-NC and WT-EMP2 3’UTR, suggesting that EMP2 was the target of miR-101-3p. Meanwhile, co-transfection with miR-101-3p and MUT-EMP2 3’UTR did not change the luciferase activity, suggesting that the miR-101-3p binding site within EMP2 was functional (Figure 7D-7E). These results suggested that up-regulating miR-101-3p inhibited EMP2 expression. To clarify the molecular mechanisms responsible for the inhibition of EMP2 expression, HNE1 cell lines were transfected with miR-101-3p mimics or inhibitors, and the expression levels of EMP2 were detected. The results showed that the miR-101-3p mimics down-regulated EMP2 expression, while miR-326 inhibitors up-regulated it. In conclusion, EMP2 is a target of miR-101-3p.

### miR-101-3p suppresses migration and cell proliferation by inhibiting EMP2 in NPC cells

**Figure 8:**
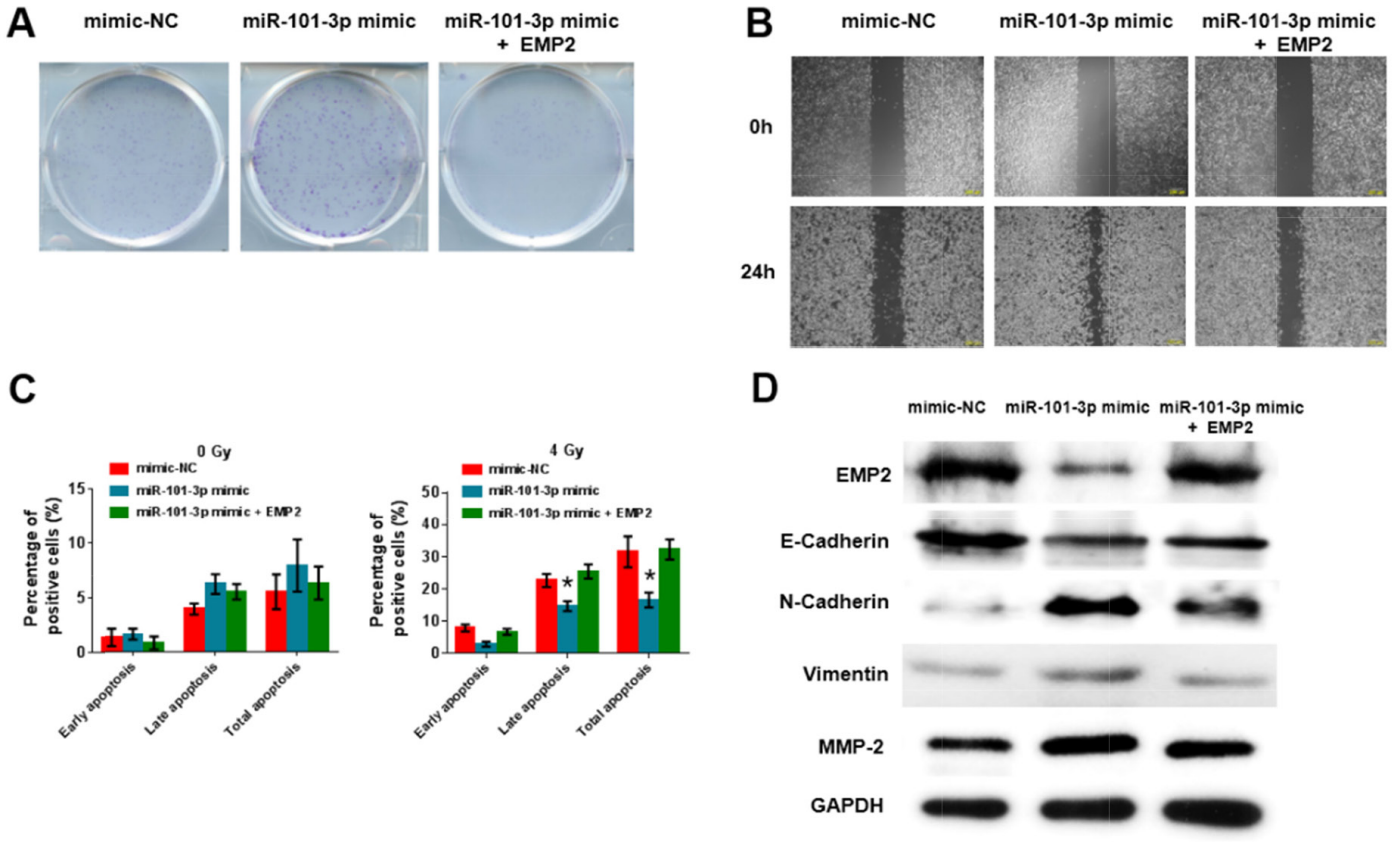
miR-101-3p suppresses migration and cell proliferation by inhibiting EMP2 in NPC cells **(A-B)** Effects of miR-101-3p on cell proliferation and migration by inhibiting EMP2 in CNE2 cells analyzed using clone formation and Wound-healing assay. The data represent the mean ± S.D. of three different experiments. *p < 0.05 versus scramble control. **(C)** miR-101-3p mimic combined with radiation treatment depresses cell apoptosis in CNE2 cells, while EMP2 promotes cell apoptosis. Flow cytometer assays were performed to examine cell apoptosis by annexin V-FITC/propidium iodide (PI) double staining. *p < 0.05. **(D)** Expression of EMP2, E-cadherin, N-cadherin, vimentin, MMP-2 and GAPDH after co-transfected CNE2 cells with EMP2 plasmid and miR-101-3p mimic. All data are represented as the mean ± S.D. from three independent experiments (**p < 0.05).

To examine if the effect of miR-101-3p on cell proliferation and migration is mediated by EMP2, we co-transfected CNE2 cells with EMP2 plasmid and miR-101-3p mimic. We demonstrated that the miR-101-3p mimic promoted the clone formation of NPC cells, while EMP2 reversed the increase in clone formation (Figure 8A). As shown in Figure 8B, wound-healing assay analysis indicated that the miR-101-3p mimic inhibited the migration of NPC cells, while EMP2 rescued this migration (Figure 8B). We further examined whether the miR-101-3p mimic combined with radiation treatment could depress cell apoptosis by annexin V-FITC/propidium iodide (PI) double staining and flow cytometry (Figure 8C). miR-101-3p mimic combined with 4 Gy radiation treatment significantly inhibited cell apoptosis in CNE2 cells; however, EMP2 promoted cell apoptosis. In addition, after CNE2 cells transfected with miR-101-3p mimic, the expression level of EMP2 was decreased and the cell migration was increased. The addition of EMP2 into CNE2 cells could reverse the tumor promotion effects of miR-101-3p mimic. Moreover, the decreased E-cadherin and up-regulated N-cadherin, vimentin and MMP-2 after miR-101-3p mimic transfection could also be inhibited by EMP2 (Figure 8D). These results indicated that the tumor-promotion function of miR-101-3p is exerted partly through the negative regulation of EMP2 in NPC.

### NEAT1 sensitized NPC to irradiation therapy *in vivo* by targeting miR-101-3p

**Figure 9:**
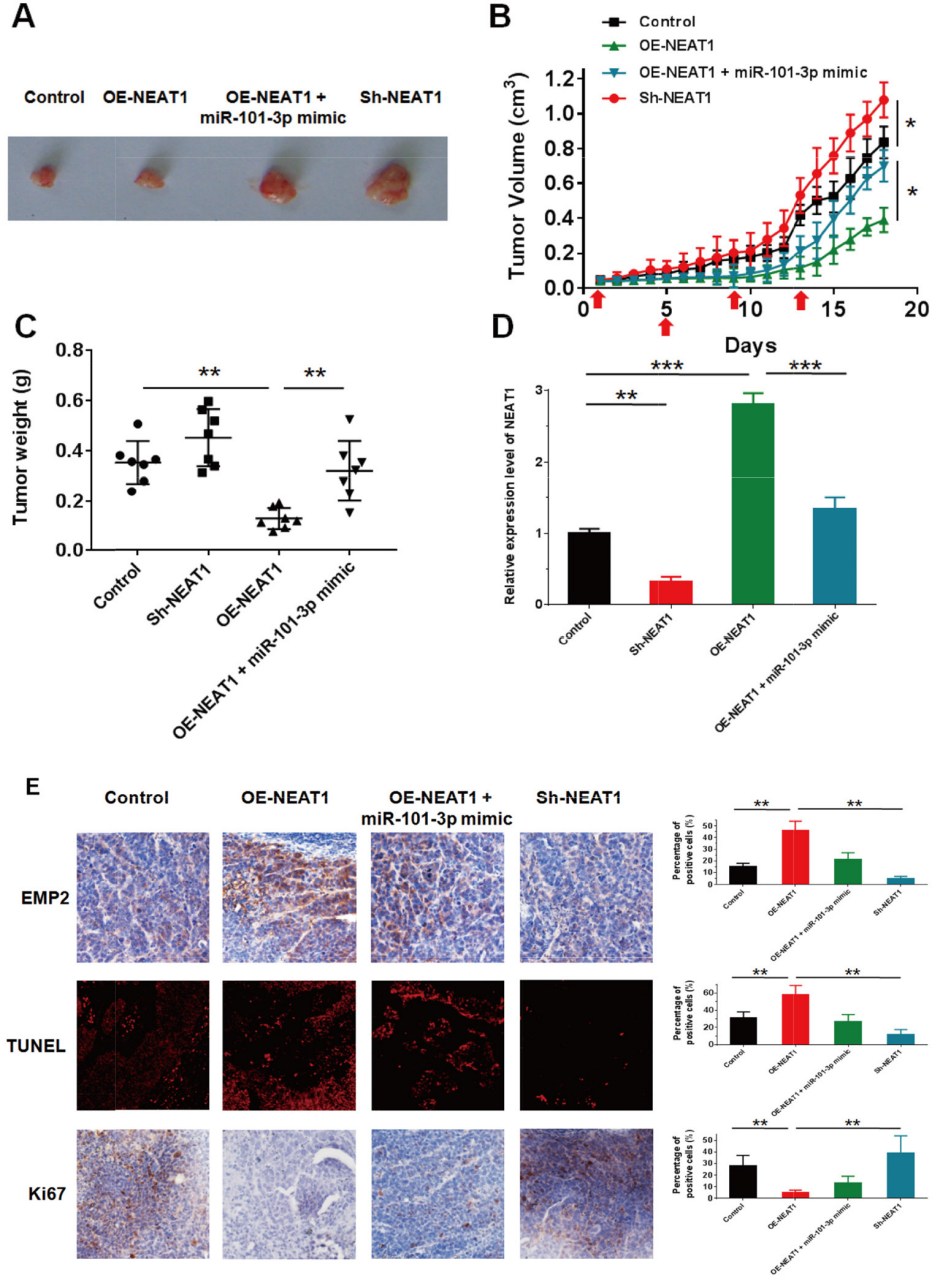
NEAT1 weaken NPC cell growth *in vivo* by inhibiting miR-101-3p **(A-C)** Tumor size, volume, and weight of subcutaneous implantation models of CNE2 cell are shown. **(D)** NEAT1 expression in tumors isolated from control, sh-NEAT1, OE-NEAT1 and OE-NEAT1+miR-101-3p mimic groups. **(E)** Immunohistochemistry of Ki67, EMP2 and immunofluorescence of TUNEL in tumors isolated from control, sh-NEAT1, OE-NEAT1 and OE-NEAT1+miR-101-3p mimic groups. Statistics were performed in triplicate. *p < 0.05, **p < 0.01, ***p < 0.001. Means ± S.D. are shown. Statistical analysis was conducted using student One-Way ANOVA test.

To verify the *in vivo* effects of NEAT on nasopharyngeal carcinoma xenograft models, CNE2 cells with OE-NEAT1, OE-NEAT1+miRNA-101-3p or Sh-NEAT1 were subcutaneously injected into nude mice, and the xenograft tumor received 4 Gy irradiation at days 1, 5, 9 and 13. The tumor volume and body weight were measured daily, all animals were sacrificed at day 18, and the tumor issues were peeled and weighed (Figure 9A-9C). Knockdown of NEAT1 significantly increased the radiation resistance of NPC *in vivo* compared with negative controls. In contrast, over-expression of NEAT1 decreased tumor growth *in vivo* compared with the negative controls, while the addition of miR-101-3p could reverse this effect (Figure 9B). By q-RT-PCR analysis, we confirmed that NEAT1 knockdown resulted in lower NEAT1 expression than in the control group. Moreover, the over-expression construct of NEAT1 showed higher NEAT1 expression than the control, and miR-101-3p could reverse the effect of NEAT1 over-expression, decreasing the expression of NEAT1 (Figure 9D). To further confirm the radiosensitization effects of NEAT1, immunohistochemical staining of Ki67 and EMP2 and TUNEL immunofluorescence were performed in different groups. The percentage of EMP2-positive cells was significantly increased in the OE-NEAT1 groups, while the addition of miR-101-3p inhibited this effect. As expected, the percentage of EMP2-positive cells significantly decreased in the Sh-NEAT1 groups. The immunohistochemical staining of Ki67 demonstrated that Ki67-positive cells were significantly reduced in the OE-NEAT1 groups, and the addition of miR-101-3p increased the Ki67(+) cells. The percentage of Ki67(+) cells increased significantly in the Sh-NEAT1 groups. The reversed results were observed in the TUNEL immunofluorescent assay of NPC tissues. The percentage of TUNEL-positive cells significantly increased in the OE-NEAT1 groups, while the TUNEL-positive cell percentage decreased when miR-101-3p was added and in the Sh-NEAT1 groups (Figure 9E). Taken together, these results revealed that the over-expression of NEAT1 enhanced the radiosensitivity of NPC cells *in vivo* by targeting miR-101-3p.

## DISCUSSION

In the current study, we determined the expression levels of NEAT1 in clinical NPC tissue samples and normal nasopharyngeal epithelial tissues and found that its expression was reduced in carcinoma tissues. In the analysis of clinical biospecimens, low NEAT1 expression was significantly associated with a higher risk of metastatic recurrence rates than high NEAT1 expression. However, these results are not completely consistent with the previously reported expression statuses of NEAT1 in NPC cell lines and tissues. These differences may be due to the collection of samples from different sources [39]. Moreover, some lncRNAs could regulate metastasis and/or irradiation-resistance in NPC, e.g., MALAT1, AFAP1-AS1, LINC01420, ROR and HNF1A-AS have been reported to promote the proliferation and metastasis of NPC cells, but LINC0086 is negatively related to the tumor size and metastasis of NPC [21, 22, 32, 34, 35, 45-48]. Recently, Fotouhi et al. and Pickard et al. reported that the lncRNAs UCA1 and GAS5 mediate the irradiation resistance of prostate cancer. However, there are no reports about the relationship between lncRNA and the irradiation of NPC. Our results confirm that the knockdown of NEAT1 did not affected cell proliferation and apoptosis but inhibited cellular migration and promoted irradiation-induced apoptosis in NPC cells, suggesting that NEAT1 played a tumor suppressor role. Notably, the over-expression of NEAT1 in NPC cells did not affect cellular proliferation but inhibited migration and promoted irradiation-induced apoptosis.

Our results demonstrate that miR-101-3p is amplified in human NPC tissues and cell lines. Some studies have also demonstrated up-regulated expression levels of miR-101-3p in several cancers, including lung cancer [49], colorectal cancer [50] and breast cancer [51, 52]. In addition, our results indicate that miR-101-3p might function by a so-called molecular sponge, or ceRNA (competing endogenous RNA), mechanism with NEAT1 and EMP2. Zhen et al. have reported that NEAT1 functions as a ceRNA to regulate c-Met expression by sponging miR-499-5p in glioma [53], and Lu et al. found NEAT1 to be targeted and regulated by miR-204 in human nasopharyngeal carcinoma [39]. We combined the predicted results from the Starbase and spongeScan databases and the next-generation sequencing of miRNAs in NPC cells after the over-expression of NEAT1 to predict the potential miRNA binding site of NEAT1. Meanwhile, our results showed that the knockdown of NEAT1 increased miR-101-3p expression. Furthermore, NEAT1 was identified as the direct target of miR-101-3p, and miR-101-3p played an oncogenic role by mediating the NEAT1 knockdown effect on NPC cells. The addition of miR-101-3p could eliminate the inhibitory effect of NEAT1 over-expression on migration and radiation resistance both *in vitro* and *in vivo*. In short, NEAT1 over-expression exerts a tumor-suppressor effect, and there is negative regulation between NEAT1 and miR-101-3p. However, the targets and functions of miR-101-3p in NPC are still unknown. Previous reports have shown that miR-101-3p acted as an oncogenic factor by down-regulating Zinc finger E-box-binding homeobox 1 (ZEB1) in hepatocellular carcinoma [54] and AMPK in triple-negative breast cancer [52]. Epithelial membrane protein-2 (EMP2) is a recently identified tumor-related protein involved in multiple biological processes, including cell migration, adhesion, proliferation and apoptosis. The expression and role of EMP2 in various tumors are complicated. To our knowledge, there is no report suggesting EMP2 could be regulated by miRNAs. Our results demonstrated that miR-101-3p directly bound EMP2 and inhibited its expression level. Furthermore, the over-expression of EMP2 could reverse the migration and radiation resistance induced by miR-101-3p. Taken together, these results indicate that the NEAT1/miR-101-3p/EMP2 axis plays an important role in the migration and radiation resistance of human nasopharyngeal carcinoma, which indicates a competitive endogenous RNA mechanism whereby nasopharyngeal carcinoma cells may develop metastasis and radiation resistance. The down-regulation of miR-101-3p induced by NEAT1 over-expression could enhance the mRNA and protein expression levels of EMP2 and then act as a tumor suppressor in NPC. However, ongoing investigations are necessary because of the possibility of other functions of NEAT1-regulated pathways. In addition, our studies suggest that NEAT1 is a potential prognostic factor for some clinically relevant endpoints of NPC.

In conclusion, the current study provides some novel insights into a ceRNA mechanism of NEAT1 regulation in the metastasis and radiation resistance of nasopharyngeal carcinoma and identifies NEAT1 as a novel prognostic biomarker for metastasis risk and radiation sensitivity. Our study discovered that NEAT1 plays a previously unexplored function by regulating miR-101-3p and EMP2. We consider NEAT1 to be highly involved in the modulation of irradiation resistance and metastatic recurrence in nasopharyngeal carcinoma. The combination of NEAT1 normalization and radiation therapy may represent a novel and promising therapeutic method within a subset of NEAT1-deficient patients with nasopharyngeal carcinoma.

## MATERIALS AND METHODS

### Tissue sample collection

NPC tissues and normal tissues were obtained from patients who were undergoing complete or partial surgical resection at Department of Radiation Oncology, Sichuan Provincial Cancer Hospital, during 2011–2015. No local or systemic treatment had been conducted in these patients before the operation. All the tissue samples were collected, immediately snap frozen in liquid nitrogen, and stored at -80°C until RNA extraction. The study was approved by the Scientific and Ethical Committee of Sichuan Cancer Hospital and Institute, China (No. 110563). Informed consent was obtained from all patients.

### Microarray data processing

The raw data of NPC tissue microarrays were retrieved and downloaded from GEO database with CEL file format, and then adjusted and normalized by using RMA method (Robust Multichip Average). The Affymettrix Expression Console and Transcriptome Analysis Console were used to process microarray data on the expression levels of mRNAs and lncRNAs. The DEGs (differential expression gene) analysis from the normalized data was used to further analysis. In all DEGs, a gene with both FDR < 0.05 and fold change ≥ 2 was considered significantly differential expressed.

### Cell lines and culture conditions

The NPC cell lines (5-8F, CNE1, CNE2, S26, HNE1, SUNE1 and HONE1) and human normal nasopharyngeal epithelial NP69 cells were obtained from Shanghai Institutes for Biological Sciences Cell Resource Center and were cultured in high glucose DMEM supplemented with 10% fetal bovine serum( Gibco, Carlsbad, CA, USA). All the cells were incubated at 37°C in a humidified incubator with 5% CO_2_.

### RNA extraction and real-time PCR

Total RNA was extracted from cells and tissues using Trizol reagent (Life Technologies Corporation, Carlsbad, CA, USA) according to the manufacturer’s instructions. The One Step SYBR® PrimeScript® PLUS RT-RNA PCR Kit (TaKaRa Biotechnology, Dalian, China) was used for the Real-Time PCR analysis to test the expression levels of NEAT1. The primers are: forward: 5′-CAGTTAGTTTATCAGTTCTCCCATCCA-3′; reverse: 5′-GTTGTTGTCGTCA CCTTTCAACTCT-3′. For the test of miR-101-3p, RNA PCR Kit (AMV) Ver.3.0 (TaKaRa Biotechnology, Dalian, China) was used, and the primers are: forward: 5’-UACAGUACUGUG AUAACUGA A-3’ and reverse: 5’-CAGUUAUCACAGUACUGUAUU-3’. For the test of EMP2, the primers are: forward: 5’-ATGGCCAGAAGCAGACATTTA and reverse: 5’-CTCACCAGACAGAAGCTGAATTA-3’. The β-actin used in this study for normalizing and the primers are: forward: 5′-CTGGGACGACATGGAGAAAA-3′; reverse: 5′- AAGGAAGGCT GGAAGAGTGC-3′. Fold changes were calculated by relative quantification (2−ΔΔCt) method.

### RNA interference by siRNA

The HNE1 cell lines were transfected by Lipofectamine 2000 (Invitrogen, Carlsbad, CA, USA) with siRNA according to the manufacture’s protocol. The sequence of NEAT1 siRNA as below: Si-NEAT1 1#: Sense GAGGGAUGAGGGUGAAGAA and antisense UUCUUCACCCU CAUCCCUC; and Si-NEAT1 1#: sense GGAGGAGUCAGGAGGAAUA and antisense UAUU CCUCCUGACUCCUCC; the negative control siRNA (si-NC) were purchased from GenePharma, Shanghai, China.

### Cell transfection

The NEAT1 knockdown (sh-NEAT1) plasmid, NEAT1 over-expression (OE-NEAT1) plasmid and the corresponding negative control (sh-NC), as well as the miR-101-3p mimic, miR-101-3p inhibitor and the corresponding negative control were synthesized (Ribobio, Guangzhou, China). The EMP2 full-length (with 3’-UTR) plasmid, EMP2 mutated plasmid (with 3’-UTR), and the negative controls were synthesized (Life technology, Carlsbad, CA, USA). The lipofectamine 2000 reagent (Life Technologies Corporation, Carlsbad, CA, USA) was used for the cells transfection according to the manufacturer’s instructions. The plasmid carrying a non-targeting sequence was used as a negative control (NC) of NEAT1 which were referred as to sh-NC. The stably transfected cells were selected by the culture medium containing 0.5 mg/ml G418 (Sigma-Aldrich, St Louis, MO, USA). After approximately 4 weeks, G418-resistant cell clones were established. MiR-101-3p mimics, inhibitors and their respective NC were synthesized (Life Technologies Corporation, MD, USA) and transfected into cells in the *in vitro* study. Because the highest transfection efficiency was occurred at 48 h, thus 72 h post-transfection was considered as the harvest time in the subsequent experiments.

### Cell proliferation

Cells were seeded into 96-well plate with a concentration of 4500 cells per well, and incubated at 37°C 1 day post infection. The number of viable cells was measured at daily intervals (hour 24, 48, 72 and 96). At each time point, 10 μl of 5 mg/ml MTT (Keygen Biotechnology, Nanjing, China) was added, and incubation was continued for 4 h. Then the medium was removed carefully and 150 μl of DMSO was added at the end of incubation. The absorbance was measured at 570 nm on the spectrophotometer. For the colony formation assay, a total of 300 cells were seeded in 6-well plates in triplicate and maintained in the complete medium for 12 days. The natural colonies were washed with PBS and fixed with 4% paraformaldehyde for 30 min at room temperature. The colonies were then stained with Crystal Violet Staining Solution for 10 min, washed with water and air-dried. The total number of colonies with more than 50 cells was counted under fluorescence microscopy.

### Quantization of apoptosis by flow cytometry

The cells were harvested and were stained with Annexin V-FITC/PI (KeyGEN Biotech, Nanjing, China) according to the instruction of the manufacturer. Then the cells were acquired by flow cytometry (FACScan, BD Biosciences, USA) and analyzed by FlowJo 7.6.1.

### microRNA sequencing and data analysis

The microRNA libraries were generated from the CNE-2 cells with OE-NEAT1 or null vector, and were sequenced on a HiSeq 4000 Sequencing platform (Illumina, San Diego, CA, USA) to identify differential expressed miRNAs. The detailed experimental procedures were followed our previously studies [55]. The cDNA was sequenced on an Illumina/Solexa sequencing platform by the Beijing Biomarker Technologies Co. Ltd. (Beijing, China).

### Wound healing assay

NPC cells were transfected with miR-101-3p mimic, si-NC, si-1#, or si1#+2#. Wounds were created in adherent cells using a 20 μL pipette tip, 24 h after transfection. The cells were then washed three times with PBS to remove any free-floating cells and debris. Medium without serum was added, and the cells were incubated under normal conditions. Wound healing was observed after 24 h under light microscopy. Representative scrape lines were photographed using digital microscopy after culture inserts were removed. Each experiment was repeated in triplicate.

### Western blotting assay

Cells were lysed with RIPA lysis buffer (Beyotime, China). Whole-cell extracts (40 μg) were then fractionated by electrophoresis through an 8% or 12% sodium dodecyl sulfate-polyacrylamide gel electrophoresis (SDS-PAGE). Gels were run at a 120 V for 2 h before transfer onto a PVDF membrane (MilliporeCorp, Billerica, MA, USA). Anti-EMP2, Anti-GAPDH, anti-E-Cadherin, anti-N-Cadherin, anti-Vimentin, anti-MMP-2 were diluted 1:1000 or 1:2000 (Proteintech Group Inc. Rosemont, USA or Abcam, USA) Following incubation with the primary antibody, blots were washed three times in TBS/Tween-20 before incubation for 1 h with goat anti-mouse or mouse anti-rabbit horseradish peroxidase conjugated antibody at a 1:10000 dilution in TBS/Tween-20 containing 5% milk. Proteins were visualized with ECL-chemiluminescent kit (ECL-plus, Thermo Scientific).

### Luciferase reporter assay

The 3’-UTR sequences of NEAT1 and EMP2 were amplified by PCR and the cloned into a pmirGlo Dual-luciferase miRNA Target Expression Vector (Promega, Madison, USA) to construct luciferase reporter vectors (NEAT1-WT and EMP2-WT, GenePharma, Shanghai, China). The miR-101-3p binding sequences in NEAT1 and EMP2 were mutated as indicated (NEAT1-MUT and EMP2-MUT). The HEK293 cells were co-transfected with the combinations of plasmids described below when they were at 50-70% confluence. A dual-luciferase reporter assay kit (Promega, Madison, USA) was used to determine the luciferase activity 48 h after transfection. Cells were harvested 48 h after transfection for luciferase assay using a luciferase assay kit (Promega, Madison, USA) according to the manufacturer’s protocol. The values were normalized to those obtained for miRNA negative control transfection. All transfection experiments were performed in triplicate.

### Xenograft models

Tumor formation was studied by establishing a xenograft model. BALB/c female nude mice (4-6 weeks old) were purchased from Beijing HFK Bio-Technology Co., Ltd (China). The animal experiments in this study were approved and reviewed by the Animal Research Committee of West China Hospital of Sichuan University. Care and handling of the animals were in accordance with the guidelines for Institutional and Animal Care and Use Committees. Mice were randomly divided into four groups with eight mice in each group. The HNE1 cells with stably transfected OE-NEAT1 or sh-NEAT1 were used to establish xenograft models. Transnfected HNE1 NPC cells (5 × 10^6^ cells/mouse) suspended in PBS solution were injected subcutaneously into the mice. The miR-101-3p mimic or saline was injected intravenous and the xenograft tumor received 4Gy irradiation at day 1, 5, 9 and 13. Tumor volumes were measured every 7 days using calipers along two major axes and calculated according to the formula V = 0.5 × L (length) × W^2^ (width). After 18 days after drug injection, all animals were sacrificed. Excised tumors were evaluated for volume and weight.

### Statistical analysis

All data were expressed as mean ± S.D. of three independent experiments, in which each assay was performed in triplicate. Statistical analysis was performed using ANOVA followed by Student’s t-test. The relationship between the expression of NEAT1 and miR-101-3p in tissues was analyzed with Pearson's correlation. Significance was defined as P < 0.05.

## Abbreviations

NPC: nasopharyngeal carcinoma
3′-UTR: 3′-untranslated regions
LncRNAs: Long non-coding RNAs
NEAT1: nuclear enriched abundant transcript 1
siRNAs: small interfering RNAs
ceRNA: competing endogenous RNA
EMP2: epithelial membrane protein 2
